# Tetanus Complicated by Dysautonomia: A Case Report and Review of Management

**DOI:** 10.1155/2021/8842522

**Published:** 2021-03-16

**Authors:** Nishant Sharma, Si Li, Metlapalli Venkata Sravanthi, Dan Kazmierski, Yichen Wang, Amit Sharma, Pragya Dhaubhadel

**Affiliations:** ^1^The Wright Center for Graduate Medical Education, 501 South Washington Avenue, Scranton, PA 18505, USA; ^2^Geisinger Community Medical Center, 1800 Mulberry Street, Scranton, PA 18510, USA

## Abstract

Tetanus is a life-threatening infectious neurological disorder that is now a rare disease due to the institution of wide-spread vaccination strategies. We present an uncommon case of generalized severe tetanus with consequent respiratory failure requiring mechanical ventilation, which was associated with dysautonomia. A 20-year-old unvaccinated female presented with neck stiffness and diffuse muscle spasms following a laceration sustained 3 weeks prior. She was admitted to the intensive care unit for mechanical ventilation and was treated with immunoglobulin, tetanus toxoid, metronidazole, and high doses of sedatives. She also developed dysautonomia, with alternating bradycardia and tachycardia, as well as fluctuating blood pressure. She was successfully extubated and discharged. We also review the epidemiology, pathophysiology, and management of tetanus and discuss dysautonomia in the setting of tetanus.

## 1. Introduction

Tetanus, a life-threatening neurological disorder involving muscle spasms caused by *Clostridium tetani*, is now a rare disease, with an annual incidence of 0.10 cases/million population in the United States [1]. We present a rare case of an unimmunized patient presenting with fulminant tetanus requiring ventilatory support, as well as severe dysautonomia. We also review the current state of disease incidence and immunization rates and summarize the management of tetanus and its complications.

## 2. Case Presentation

A 20-year-old unvaccinated female with a history of substance use disorder presented to the emergency department with neck stiffness and diffuse muscle spasms for three days. Her symptoms had started with neck and back stiffness three days prior and had progressed to involve other muscle groups, causing an inability to speak, swallow, or turn her head, as well as severe muscle spasms of the torso and all extremities. She had, of note, sustained a laceration on her wrist 3 weeks prior and had not sought medical evaluation. Furthermore, she was an active intravenous drug user and admitted to injecting heroin as recently as 2 days prior to presentation. Further elaboration of medical history revealed that she had never received any vaccinations because her mother “did not believe in them.”

Physical examination revealed a heart rate of 85 beats min^−1^, blood pressure of 137/65 mm Hg, respiratory rate of 23 breaths min, and a temperature of 99.5 F. She appeared ill and was in severe distress; notable findings were trismus, clenched jaw, drooling, and diffuse muscular rigidity. Wound site contractions were noted around a 5-centimeter-long laceration on the right wrist. Cardiovascular, respiratory, abdominal, and sensory examination was unremarkable. Laboratory work-up was notable for leukocytosis. Urine toxicology was positive for opiates. Computed tomography of the neck was grossly unremarkable for acute pathology.

The combination of her presentation, recent injury, and unimmunized status prompted the clinical diagnosis of generalized tetanus with subsequent administration of human tetanus immunoglobulin and tetanus toxoid and the initiation of metronidazole. The intravenous agents were administered in the antecubital vein of the same arm which had sustained the laceration. She was admitted to the intensive care unit and placed in a room with minimal auditory and visual stimuli. Diazepam, baclofen, fentanyl, and magnesium were initiated for control of muscle spasms. Shortly after admission, she developed progressive trismus and airway compromise, leading to nasotracheal intubation and initiation of mechanical ventilation. This was followed by percutaneous tracheostomy in anticipation of prolonged ventilation. Sedation proved to be challenging, requiring high doses of intravenous propofol (65 mcg/kg/min), fentanyl (200 mcg/hr), and midazolam (7 mg/hr) infusions. However, with continued supportive care, she was weaned off mechanical ventilatory support on ICU day 7. She continued to have a complicated hospital course, which was most notable for hemodynamics, with alternating tachycardia and bradycardia, and fluctuating blood pressure, attributable to autonomic dysfunction due to tetanus. Her hemodynamics stabilized with continued magnesium supplementation and supportive care. Fiberoptic laryngoscopy confirmed satisfactory airway patency, and her tracheostomy was decannulated. She was discharged after a 26-day-long hospital course, and a catch-up immunization schedule was arranged. The patient continued to have residual neck stiffness upon follow-up 6 months after discharge, which was treated conservatively with muscle relaxants.

## 3. Discussion

Tetanus is now a rare disease in the developed world, owing to the institution of universal vaccination policies. 23 cases of tetanus were reported in 2018 to the National Notifiable Diseases Surveillance System (NNDSS), maintained by the Center for Disease Control (CDC) [2]. Vaccination coverage, however, while high, remained incomplete—in 2017, only 83.2% of children aged 19-35 months living in the US received the full dose of the tetanus vaccine [3]. A review of vaccination coverage among adults in 2016 showed that merely 62.2% of adults over 19 years of age reported having received any tetanus toxoid containing vaccination during the preceding 10 years [4]; this figure was even lower in adults over 65 years of age: 58% [4]. Given the prevalent age of increasing vaccine skepticism, it is important that health care providers be familiar with the management of tetanus, which if left untreated has a case mortality rate approaching 100% [5]. The purpose of this presentation is to discuss the management of generalized tetanus in a community setting, in a case which was complicated by a history of substance abuse and the development of autonomic dysfunction.

Tetanus is caused by *Clostridium tetani*, a gram-positive, spore-forming, obligate anaerobe. Its spores are ubiquitous and can be found in soil, as well as the intestinal tract and feces of humans and animals. They are resilient and are incompletely destroyed by boiling. They can achieve entry into the body through disruption in the continuity of skin and, under favorable conditions, such as the anaerobic environment of devitalised tissue, germinate into toxin-producing bacteria.

The bacterium then produces two toxins: tetanolysin and tetanospasmin [6]. Tetanolysin is hypothesized to play a role in the destruction of local tissue and optimization of conditions for bacterial proliferation. Tetanospasmin disrupts synaptic neurotransmission by preventing neurotransmitter release from affected neurons. Local nerve terminals at the site of the wound are first affected, followed by hematogenous spread and retrograde axoplasmic transport, the end result being potential diffuse involvement of the nervous system. Inhibitory neurons are most affected, with the consequent inhibition of the release of gamma-aminobutyric acid (GABA) and glycine, both inhibitory neurotransmitters. It is the loss of inhibition and consequent uncontrolled efferent discharges, which lead to the syndrome of widespread muscle spasms and autonomic dysfunction that constitutes the clinical presentation of tetanus.

The incubation period of the disease is anywhere between 3 and 21 days following infection [5]. There are three categories of clinical presentation:

1. Localized tetanus, typically seen in infections with a low toxin load, where spasms and rigidity are limited to the area around the wound
2. Cephalic tetanus, a rare form of tetanus associated with cranial infections, which can present as cranial nerve palsies
3. Generalized tetanus, characterized by widespread rigidity and spasms throughout the body

Early symptoms of the disease include neck stiffness and difficulty opening the mouth (lockjaw), caused by masseter muscle spasm. Facial muscles can be progressively involved, causing the distinctive facial expression associated with tetanus, risus sardonicus. Involvement of the trunk follows, with severe spasms of the back musculature, causing opisthotonus, the backward arching of the neck and back, and increased muscle tone and tonic spasms in the extremities. Autonomic involvement may also be seen and is an indicator of a poor prognosis. The sympathetic system is usually affected, with resultant tachycardia, hypertension, and vasoconstriction, but bradycardia and hypotension may also be seen. Other autonomic manifestations include increased salivation and secretions and gastric dysmotility.

There are many proposed grading systems, but the system proposed by Ablett is most commonly used (Table 1) [6].

The management of tetanus has its basis in three principles: first, the prompt neutralization of unbound toxin with the intramuscular injection of 3,000-6,000 units of human tetanus immunoglobulin. Second, the prevention of further toxin production by the administration of metronidazole, which is the antibiotic of choice. Penicillin is active against *C. tetani* but is a GABA antagonist and may be associated with convulsions [7]. Other antibiotic options include doxycycline, clindamycin, vancomycin, macrolides, and chloramphenicol. Additionally, local debridement of the wound to eradicate spores is of paramount importance.

Finally, the effect of tetanospasmin that has already achieved entry into the neuronal system must be neutralized. The patient must be placed in a room with minimal visual and auditory stimulation to avoid provoking muscle spasms. The mainstay of pharmacological treatment of diffuse muscle spasms, as seen with generalized tetanus, is administration of benzodiazepine, which increases gamma aminobutyric acid (GABA) [8]. Diazepam has been used with success but can cause prolonged sedation as well as anion gap metabolic acidosis at high doses. Midazolam appears to have fewer risks and can be used as a continuous infusion. When benzodiazepine-induced sedation proves to be inadequate, neuromuscular blockade can be instituted with agents such as pancuronium. Pancuronium inhibits the reuptake of catecholamine and can worsen autonomic instability; vecuronium is a shorter-lasting alternative which is less likely to cause cardiovascular adverse effects. Propofol, dantrolene, and intrathecal baclofen have all been reported to be of utility [9–11]. Dexmedetomidine, which has antisympathetic properties, is useful as an adjunct for sedation [8].

Autonomic dysfunction in association with generalized tetanus portends a poor prognosis [12]. Dysautonomia is not an uncommon complication of tetanus; one cross-sectional study found an incidence of 11.8% among patients who were hospitalized for tetanus [13]. Its cardiovascular manifestations are varied and range from “autonomic crises” characterized by episodes of profound hypertension and tachycardia to bradycardia, arrhythmias, and hypotension. The hyperkinetic circulatory state observed with autonomic storms has been attributed to increased sympathetic activity; there exists an elevated basal level of norepinephrine, with spikes in epinephrine during times of autonomic storms [14]. Possible mechanisms of bradycardia, as seen in our patient, are sudden withdrawal of catecholamines or increased vagal tone. The management of these cardiovascular aberrations includes the administration of combined *α* and *β* adrenergic blocking agents, such as labetolol. *β*-Adrenergic blocking agents should be avoided because of their potential to cause hypotension, pulmonary edema, and sudden death. There have been reports of atropine being used to alleviate cardiovascular instability [15]. Clonidine, a centrally acting *α*-2 agonist, reduces sympathetic outflow and may be useful in the control of sympathetic overactivity in tetanus, as demonstrated by Sutton et al. [16]. Semipermanent cardiac pacing has been shown to be useful in the management of severe bradycardia in generalized tetanus [17]. Magnesium is a presynaptic neuromuscular blocker, blocks catecholamine release from nerves, and reduces the responsiveness of receptors to catecholamines. Magnesium sulphate has been successfully used to reduce autonomic disturbance and muscle spasms [18]. One study found some success with the use of epidural blockade with bupivacaine and sufentanil, which resulted in a significant reduction in blood pressure fluctuation [19].

A special note must be made about the occurrence and management of tetanus in heroin users, such as our patient. This population appears to be at a higher risk of contracting tetanus, not only because of the potential for contaminated intravenous access sites but also because quinine, which is used to dilute heroin, may support the growth of *C. tetani* [20]. In addition, pharmacological treatment, which is a critical component of the treatment of generalized tetanus, is challenging in these patients. A high level of tolerance to sedatives is frequently encountered, necessitating the administration of high doses of multiple agents to achieve target levels of sedation. One study found nonsignificant greater total requirements for sedation and analgesia in patients with a history of substance abuse, compared to those without [21]. This patient required high doses of fentanyl, propofol, and midazolam concurrently for adequate relief of muscle spasms.

## 4. Conclusion

In conclusion, we present the clinical presentation and management of a severe case of tetanus complicated by autonomic dysfunction and respiratory failure requiring mechanical ventilation. We review the pathogenesis and management of this deadly disease. Tetanus is a rare disease, the management of which intensivists must be familiar with, given the prevalent era of growing vaccine skepticism and incomplete immunization.

## Figures and Tables

**Table 1:** 

Grade	Severity	Symptoms
1	Mild	Mild to moderate trismus; general spasticity; no respiratory embarrassment; no spasms; little or no dysphagia.
2	Moderate	Moderate trismus; well-marked rigidity; mild to moderate but short spasms; moderate respiratory embarrassment with an increased respiratory rate greater than 30; mild dysphagia.
3	Severe	Severe trismus; generalized spasticity; reflex prolonged spasms; increased respiratory rate greater than 40; apnoeic spells; severe dysphagia; tachycardia greater than 120.
4	Very severe	Grade III and violent autonomic disturbances involving the cardiovascular system. Severe hypertension and tachycardia alternating with relative hypotension and bradycardia, either of which may be persistent.
